# Pharmacological Effects of Two Novel Bombesin-Like Peptides from the Skin Secretions of Chinese Piebald Odorous Frog (*Odorrana schmackeri*) and European Edible Frog (*Pelophylax kl. esculentus*) on Smooth Muscle

**DOI:** 10.3390/molecules22101798

**Published:** 2017-10-23

**Authors:** Xiaowei Zhou, Chengbang Ma, Mei Zhou, Yuning Zhang, Xinping Xi, Ruimin Zhong, Tianbao Chen, Chris Shaw, Lei Wang

**Affiliations:** 1 Department of Nutrition, Henry Fok School of Food Science and Engineering, Shaoguan University, Shaoguan 512005, China; xzhou06@qub.ac.uk; 2 Natural Drug Discovery Group, School of Pharmacy, Queen’s University, Belfast BT9 7BL, Northern Ireland, UK; c.ma@qub.ac.uk (C.M.); m.zhou@qub.ac.uk (M.Z.); yzhang25@qub.ac.uk (Y.Z.); t.chen@qub.ac.uk (T.C.); chris.shaw@qub.ac.uk (C.S.); l.wang@qub.ac.uk (L.W.)

**Keywords:** bombesin-like peptide, frog, skin secretion, smooth muscle

## Abstract

Bombesin-like peptides, which were identified from a diversity of amphibian skin secretions, have been demonstrated to possess several biological functions such as stimulation of smooth muscle contraction and regulation of food intake. Here, we report two novel bombesin-like peptides, bombesin-OS and bombesin-PE, which were isolated from *Odorrana schmackeri* and *Pelophylax kl. esculentus*, respectively. The mature peptides were identified and structurally confirmed by high performance Scliquid chromatography (HPLC) and tandem mass spectrometry (MS/MS). Subsequently, the effects of these purified chemically-synthetic peptides on smooth muscle were determined in bladder, uterus, and ileum. The synthetic replications were revealed to have significant pharmacological effects on these tissues. The EC_50_ values of bombesin-OS for bladder, uterus and ileum, were 10.8 nM, 33.64 nM, and 12.29 nM, respectively. Furthermore, compared with bombesin-OS, bombesin-PE showed similar contractile activity on ileum smooth muscle and uterus smooth muscle, but had a higher potency on bladder smooth muscle. The EC_50_ value of bombesin-OS for bladder was around 1000-fold less than that of bombesin-PE. This suggests that bombesin-OS and bombesin-PE have unique binding properties to their receptors. The precursor of bombesin-OS was homologous with that of a bombesin-like peptide, *odorranain*-BLP-5, and bombesin-PE belongs to the ranatensin subfamily. We identified the structure of bombesin-OS and bombesin-PE, two homologues peptides whose actions may provide a further clue in the classification of ranid frogs, also in the provision of new drugs for human health.

## 1. Introduction

Bombesin, a 14-amino acid peptide (QQRLGNQWAVGHLM-NH_2_), was originally isolated from the skin of the European toad, *Bombina bombina* [1]. Three bombesin-like peptides, gastrin-releasing peptide (GRP), neuromedin B peptide (NMB) and neuromedin C peptide (NMC), were successively identified from porcine non-antral gastric tissue and porcine spinal cord [2,3,4]. There are a large number of bombesin peptides and their precursor cDNAs known, which has been confirmed from skin secretions of various species [5,6]. Normally, these peptides have a pyroglutamyl residue at the N-terminal and an amidated residue (usually a Met) at the C-terminal, like the original bombesin peptide. Apart from this, they are also widely-distributed in terms of both mammalian neural and endocrine cells. Bombesin-like peptides, like many other active peptides, are synthesized as larger protein precursors that are enzymatically converted to their mature forms.

Bombesin-like peptides, as a family of neuro-endocrine peptides, have been divided into three groups including the bombesins, the ranatensins and the phyllolitorins [7,8,9]. Each subfamily is characterized by a common amino acid near its C-terminal. They combine with G-protein coupled receptors for regulating physiological processes. It has been demonstrated that there are five subtypes of G-protein coupled receptors in the bombesin-like receptor family, which include the NMB receptor (BB1-R), the GRP receptor (BB2-R), bombesin receptor subtype-3 (BB3-R), bombesin receptor subtype-4 (BB4-R) and BB3.5-R [10,11,12]. However, only BB1-R, BB2-R and BB3-R are found in mammalian tissues. These mammalian receptors are mainly distributed in the central nervous system (CNS) and gastrointestinal (GI) tract [13].

Scientists have found that bombesin-like peptides are involved in central functions which include regulation of food intake [14], regulation of anxiety and fear–related behaviour [15], regulation of temperature [16], and integration of stress and memory [17]. Hence, it is particularly important to study bombesin-like peptides in fields of obesity, promoting spontaneous delivery, and reducing postpartum haemorrhage, and also in the treatment of nervous system diseases.

Here, we report two natural bombesin-like peptides, bombesin-OS and bombesin-PE, which were first identified from the skin secretions of *Odorrana schmackeri* and *Pelophylax kl. esculentus*, respectively. Their pharmacological activity was tested in rat smooth muscles including bladder, uterus and ileum. Both were demonstrated to cause significant contractile effects on these three tissues.

## 2. Results

### 2.1. Molecular Cloning of Bombesin-OS and Bombesin-PE Precursor-Encoding cDNAs

**Figure 1:**
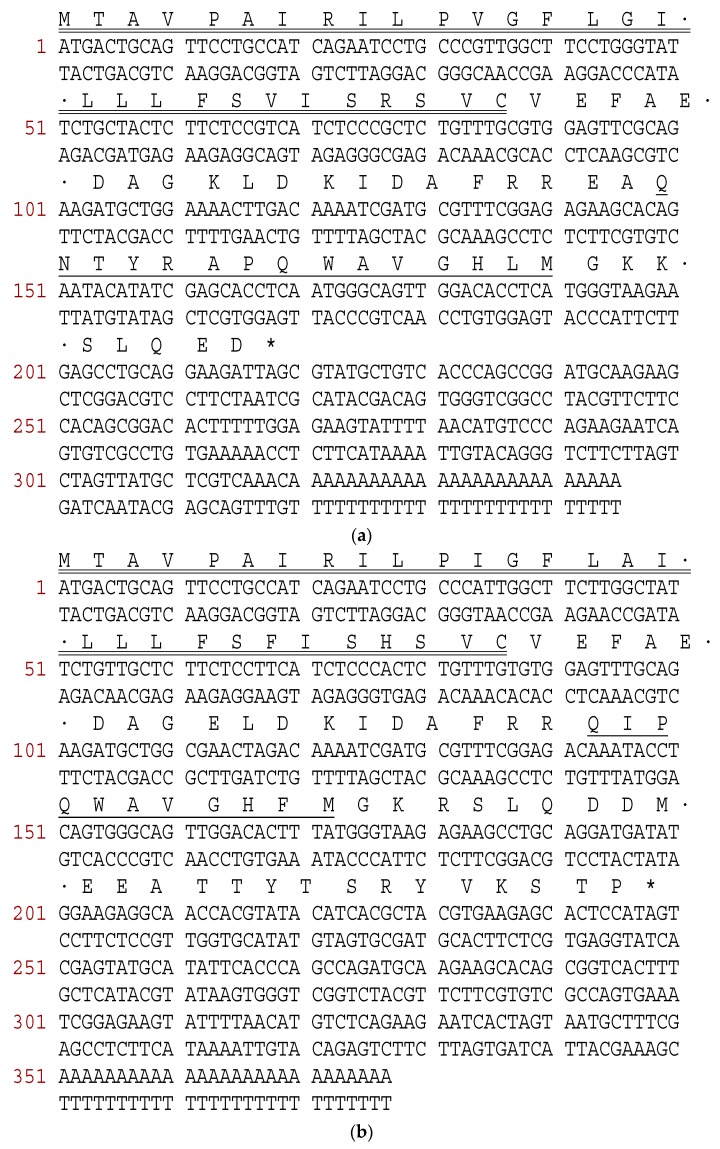
The sequences of cDNAs encoding bombesin-OS and bombesin-PE precursors. (**a**) Nucleotide and corresponding translated open-reading frame amino acid sequence of precursor cDNA cloned from the Chinese piebald odorous frog cDNA encoding bombesin-OS; (**b**) Nucleotide and corresponding translated open-reading frame amino acid sequence of precursor cDNA cloned from the skin secretion of European edible frog cDNA encoding bombesin-PE. Putative signal peptides are double-underlined, mature peptides are single-underlined and stop codons are marked by asterisks.

**Figure 2:**
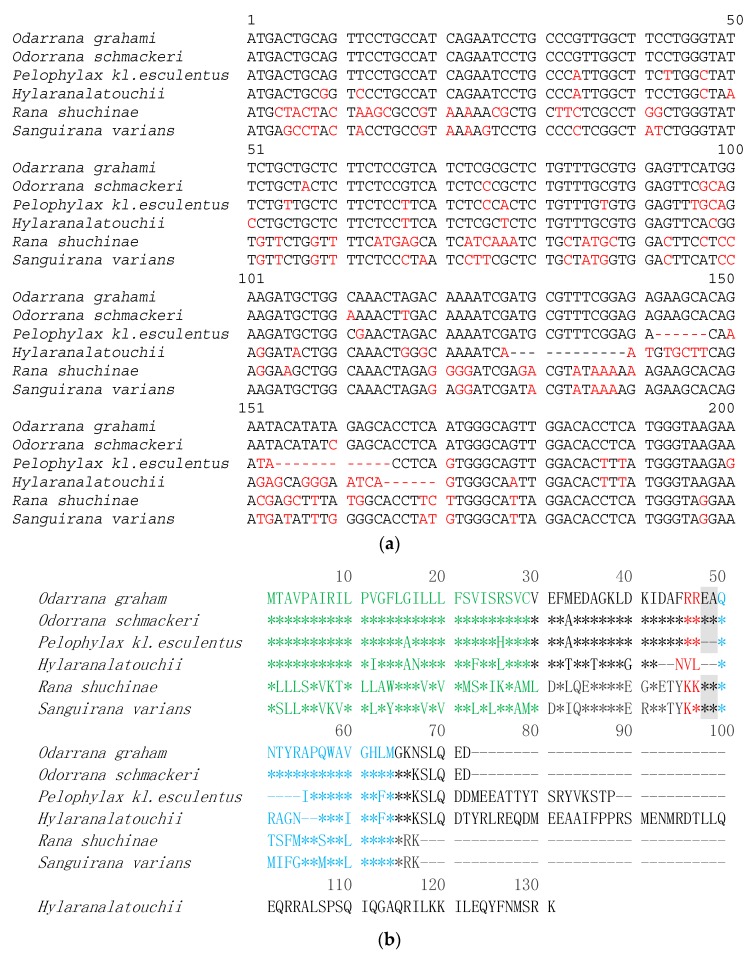
Alignments of partial nucleotides and translated open-reading frame amino acids sequences of bombesin peptides from different species of the Ranidae family. (**a**) Partial nucleotides sequences of bombesin peptides from different species of the Ranidae family; (**b**) The translated open-reading frame amino acids sequences of bombesin peptides from different species of the Ranidae family. The different nucleotides in (**a**) are labelled in red. The sequences of mature peptides in (**b**) are labelled in blue. The sequence of signal peptide are labelled in green. Stars (*) indicate the identical amino acid residues. The processing sites of the precursor for releasing mature peptides are labelled by red. The second possible processing sites are in shadow. Gaps (dashed line) were introduced to optimise the identities.

Bombesin-OS and bombesin-PE precursors were repeatedly cloned from the cDNA library constructed from the skin secretion of *Odorrana schmackeri* and *Pelophylax kl. esculentus*, respectively. The nucleotide sequences between open-reading frames of the cloned precursor transcripts and their related translated amino acid sequences are shown in Figure 1. Specifically, the bombesin-OS precursor had 72 amino acids, which include a signal peptide (29 amino acids), an N-terminal extension peptide followed by a typical putative propeptide convertase processing site (-RR-), a mature peptide (15 amino acids), C-terminal acidic extension peptide containing a further convertase processing site (-KK-), and a glycyl residue amide donor. However, the bombesin-PE precursor consisted of 88 amino acids, including a signal peptide (29 amino acids), an N-terminal extension peptide, a mature peptide (11 amino acids), C- terminal extension peptide containing a further convertase processing site (-KR-), and a glycyl residue amide donor. BLAST analysis of bombesin-OS and bombesin-PE using the NCBI database, revealed that Bombesin-OS has 100% identity with a bombesin-like peptide from *Odarrana grahami* (Figure 2b). Meanwhile, bombesin-PE was demonstrated to belong to a typical bombesin subfamily, the ranatensins. The precursor sequence alignment of bombesin-OS and bombesin-PE with homologues from other ranid frogs is shown in Figure 2b [18,19,20,21,22]. The nucleotide sequence of the cDNA encoding bombesin-PE and bombesin-OS precursors have been made available in the European Molecular Biology Laboratory (EMBL) Nucleotide Sequence Database under the accession codes, MF784811 and MF784812.

### 2.2. Identification and Structural Characterisation of Bombesin-OS and Bombesin-PE

**Figure 3:**
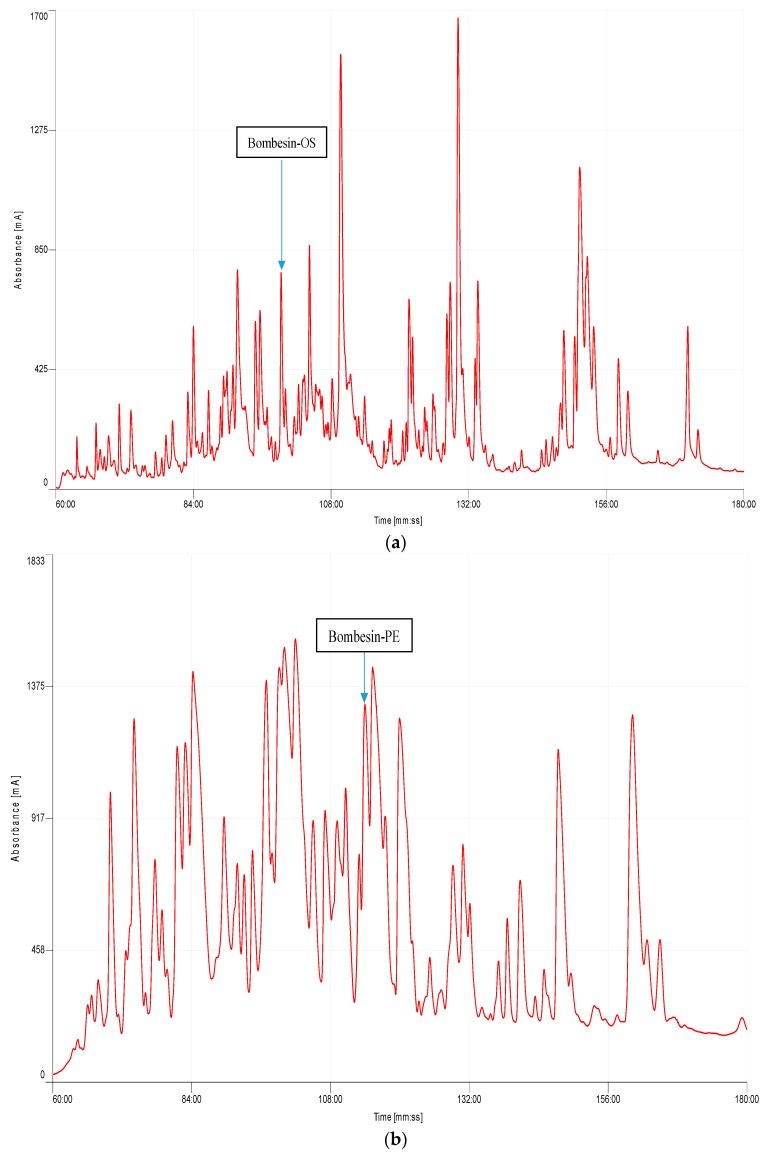
Reverse phase HPLC chromatogram of frog skin secretions. (**a**) HPLC Chromatogram of Chinese piebald odorous frog (*Odorrana schmackeri*); (**b**) HPLC chromatogram of European edible frog (*Pelophylax kl. esculentus*) skin secretion.

**Figure 4:**
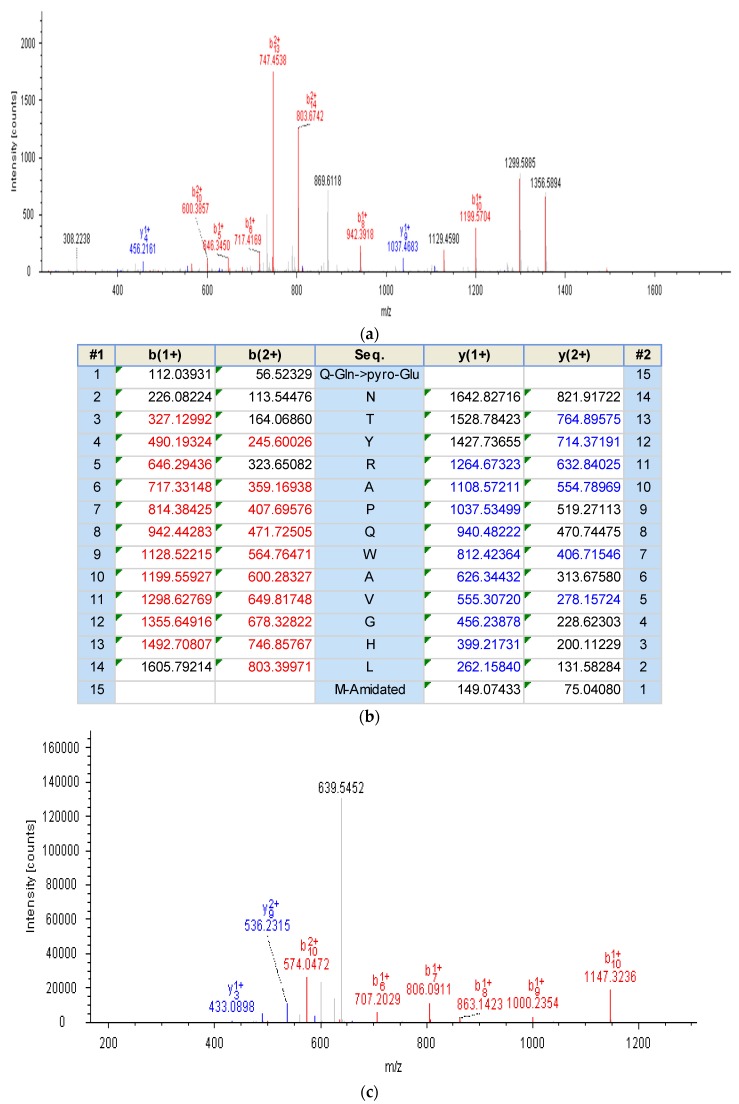

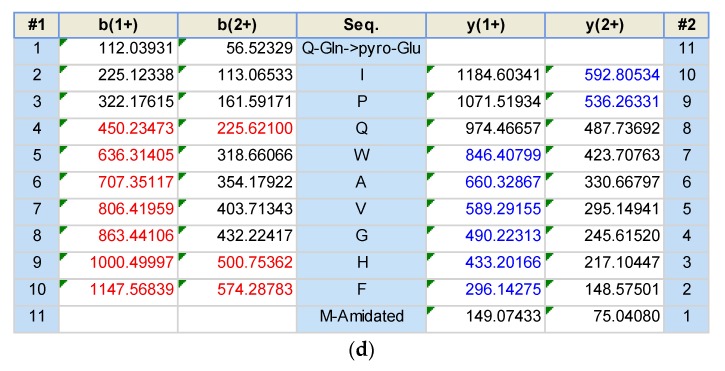
Liquid chromatography coupled tandem mass spectrometry (LC/MS/MS) spectra and predicted b- and y-ion MS/MS fragment ion series of bombesin-OS and bombesin-PE. (**a**) Annotated tandem mass spectrometry (MS/MS) fragmentation spectrum of bombesin-OS; (**b**) Predicted singly and doubly charged b- and y-ions arising from MS/MS fragmentation of bombesin-OS. The observed b- and y-ions are showed in red and blue, respectively; (**c**) Annotated MS/MS fragmentation spectrum of bombesin-PE; (**d**) Predicted singly and doubly charged b- and y-ions arising from MS/MS fragmentation of bombesin-PE. The observed b- and y-ions are showed in red and blue, respectively.

Both bombesin-OS and bombesin-PE were identified in the skin secretions of *Odorrana schmackeri* and *Pelophylax kl. esculentus*, respectively (Figure 3). A component (Figure 3a) with a mass of 1754.5 Da (Figure 4a) and a component (Figure 3b) with a mass of 1295.39 Da (Figure 4c) were found to possess considerable smooth muscle contractile activity. The primary structures of these peptides were determined by tandem mass spectrometry (MS/MS) fragmentation sequencing (Figure 4). 

### 2.3. Pharmacological Effects of Bombesin-OS and Bombesin-PE on Smooth Muscle

**Figure 5:**
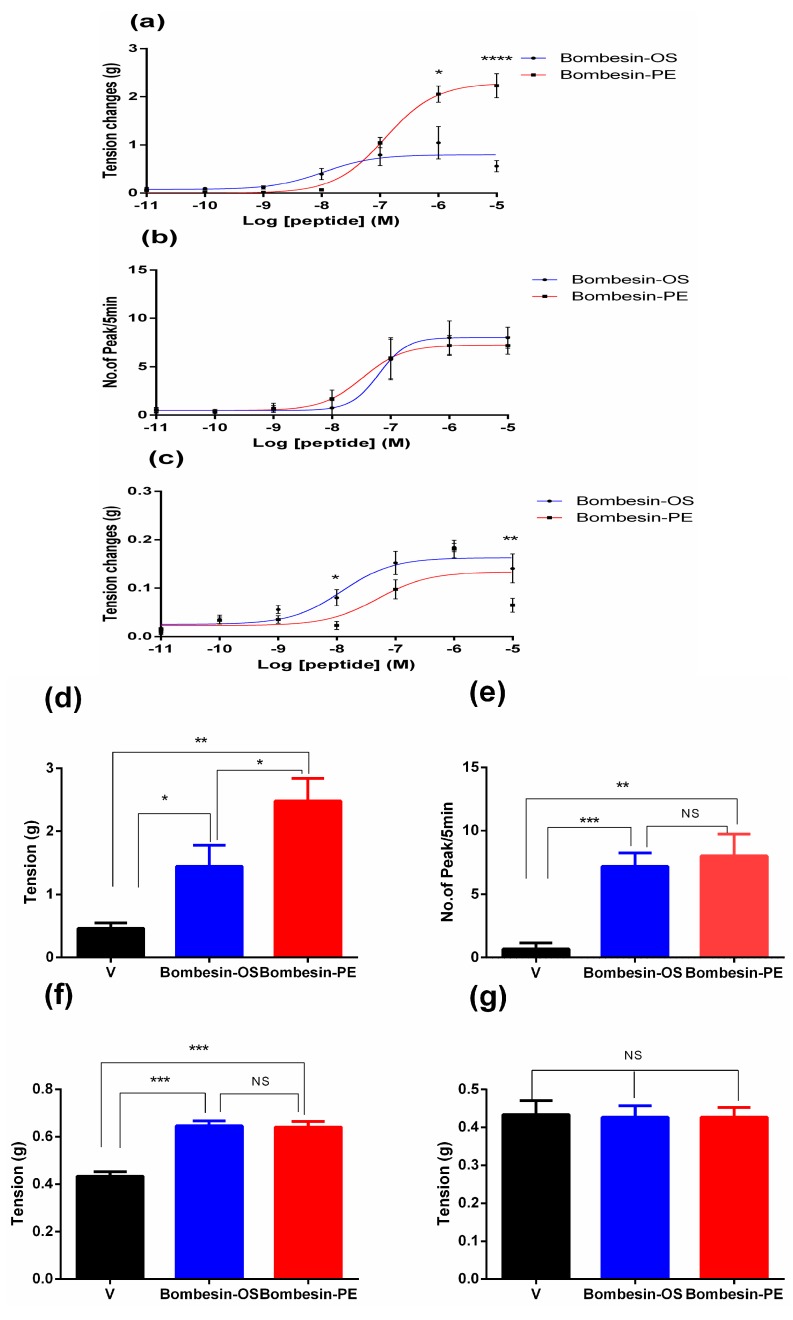
Comparison of myotropic effects of synthetic bombesin-OS and bombesin-PE on isolated rat urinary bladder, uterus, and ileum smooth muscles. Dose response curves of bombesin-OS and bombesin-PE actions on the smooth muscle preparations from (**a**) rat bladder (observed power^b^ = 0.998); (**b**) rat uterus (observed power^b^ = 1.000); and (**c**) rat ileum (observed power^b^ = 1.000). Pharmacological effects of bombesin-OS and bombesin-PE, at 1 µM, on the smooth muscle preparations from (**d**) rat bladder [F(2,24) = 3.949); observed power^b^ = 0.978]; (**e**) rat uterus [F(2,24) = 8.047; observed power^b^ = 0.973]; (**f**) rat ileum [F(2,24) = 8.388); observed power^b^ = 1.000] and (**g**) rat artery [F(2,23) = 0.01791; observed power^b^ = 0.875]. Data represent means ± SEM of three independent experiments with nine replicates; NS represents no significant difference; V represents vehicle control. **** *p* < 0.0001, *** *p* < 0.001, ** *p* < 0.01 and * *p* < 0.05 indicate significant difference.

The purified peptides were used in assessment of pharmacological activity on rat bladder, uterus, and ileum smooth muscles. Both bombesin-OS and bombesin-PE possessed significant contractile activity on rat bladder, uterus, and ileum (Figure 5). Specifically, the EC_50_ values of bombesin-OS on rat bladder, uterus, and ileum were 10.82 nM, 33.64 nM, and 12.29 nM, respectively. The EC_50_ values of bombesin-PE were 10.65 µM, 56.82 nM, and 43.1 nM on bladder, uterus, and ileum, respectively. Both bombesin-OS and bombesin-PE showed no contractile activity on rat artery. Furthermore, compared with bombesin-PE, the EC_50_ value of bombesin-PE (10.65 µM) on rat bladder was nearly 1000 times greater than that of bombesin-OS (10.82 nM), and therefore bombesin-OS might be cause a more potent contractile effect on rat bladder. However, the contractile activity of bombesin-OS on ileum and uterus smooth muscles was essentially the same to bombesin-PE. The results of post hoc analysis of contractile activity of isolated peptides are shown in Table 1.

## 3. Discussion

Amphibian skin secretion contains many bioactive compounds such as proteins, peptides, alkaloids, and steroids. Recently, there has been more research focusing on the bioactive compounds in the skin secretions of amphibians because of their broad range of pharmacological properties. However, amphibians are suffering from threats caused by climate change and human encroachment. A large proportion of these compounds include neuroactive peptides like bombesin. As explained previously, bombesin mediates its functions through specific receptors. Additionally, bombesin and their receptors are widely distributed in the periphery and CNS, and are associated with various functions like food intake, pain, stress, and fear responses [23]. Henceforth, it will be meaningful for scientists to study the physiological and pathological aspects of bombesin relationships with receptors in amphibians. In this study, the initial purpose was the “shotgun cloning” to obtain novel peptides by using degenerate primers against bombesin-like peptides. This was followed by peptide synthesis and pharmacological assessment of the smooth muscle contractile activity of these synthetic peptides and their relationships to cognate receptors. 

Bombesins, are widely found in the skin secretions of amphibians, including in typical water frogs such as the marsh frog, *Rana ridibunda* [24]. They contain an active octapeptide motif, -QWAXGXXM-, at the C-terminal, which is helpful for binding to BB1 and BB2 receptors [25]. This study is the first report to identify bombesin in the skin secretion of *Odorrana schmackeri*. Additionally, although a bombesin-related peptide has been found in the skin secretion of *Pelophylax kl. esculentus* [26], our study identified a novel bombesin propeptide from *Pelophylax kl. esculentus*, and confirmed the mature peptide in the skin secretion. Moreover, the present study demonstrated that the mature peptides were successfully identified in skin secretion after the mRNAs of bombesin-OS and bombesin-PE were cloned from a skin secretion cDNA library, using the described “shotgun” cloning approach. The overall structures of precursors of bombesin-like peptides from different species of the Ranidae family are illustrated in Figure 2b. Among these, there were significant differences in the number and in the processing patterns of these bombesin-like peptides and their precursors, which consisted of 72 (*Odorrana schmackeri* and *Odorrana graham*), 88 (*Pelophylax kl. esculentus*), 131 (*Hylaranalatouchii*) and 67 (*Rana shuchinae* and *Sanguirana varians*) amino acids, respectively. Moreover, the enzymatic processing sites in the N-terminal parts as well as the catalytic sites in the C-terminal parts were shown to be different between the bombesin-like peptides (Figure 2b). The N-terminal processing sites are always -RR-, -KR-, -EA-, and -KK-, while the C-terminal processing sites are -KK-, -KN-, and -RK-. Additionally, like other bombesin-like peptides from amphibians [21], there is an amidation of the C-terminal amino acid residue in both bombesin-OS and bombesin-PE, which may improve the stability of peptide in vivo. In all bombesin-like peptides found in Rana species, there exists a pyroglutamyl residue in the N-terminal, comprising the N-terminal catalytic sites of bombesin-like peptides. Some also contain processing sites (-EA-) in the precursor (Figure 2) but these are not found in the precursor of bombesin-PE. It has been suggested that this dipeptide processing site may not be beneficial for the formation of pyrocarbamylation. Our data is consistent with the conclusion that an N-terminal glutamine provides the N-terminal pyroglutamyl residue and a C-terminal glycine provides the amide for the C-terminal amide [20]. The differences of catalytic sites may be attributed to alterations in the posttranslational modification [27]. These data suggest that the signal peptide domain of precursor as well as the cleavage sites of amphibian bombesin-like peptides can be a measure for the classification and evolution of animal species as suggested by Li et al. [21].

The sequences of these two novel peptides have characteristics of an N-terminal pyroglutamic acid, an internal motif -QWAVGXM-, and a C-terminal amide, which are highly-conserved in other bombesins from amphibians [19,20]. It is interesting to note that a degenerate primer from the *Odorrana* species was used to obtain the cDNA of bombesin-OS precursor. However, unexpectedly, the cDNA of bombesin-PE precursor, which comes from *Pelophylax kl. esculentus*, could also be cloned using the same degenerate primer from the *Odorrana* species. Specifically, both *Odorrana schmackeri* and *Pelophylax kl. esculentus* showed low homology compared with the precursors of *Rana shuchinae* and *Sanguirana varians*. It was noted that *Odorrana schmackeri*, *Pelophylax kl. esculentus* and *Odorrana grahami* are mainly distributed in southern and central China, respectively, while *Rana shuchinae*, *Pelophylax kl. esculentus* and *Sanguirana varians* are distributed in south-western China, Europe, and the Malay Archipelago, and it could explain that novel peptides sequences can be obtained from different *Rana* species living in the same area [28] and this could be used to investigate the evolution of amphibians as well. 

Both bombesin-PE and bombesin-OS showed similar contractile activity in uterus smooth muscle and ileum smooth muscle. Since the bladder expresses the NMB-preferring subtype receptor while the ileum and uterus express the GRP-preferring subtype of bombesin receptor [29,30], it was suggested that bombesin-OS and bombesin-PE bind to the GRP receptor in the ileum and uterus but to the NMB receptor in the bladder. However, bombesin-OS displayed more potent contractile activity in bladder smooth muscle compared with bombesin-PE. Comparing the primary structures of these two peptides, they share a similar sequence (-PQWAVGHXMNH_2_) in the C-terminal, while the N-terminal of bombesin-PE (-pGlu-QI-), is shorter than that of bombesin-OS which has more amino acids (-pGlu-QNTYRA-). This suggested that the longer stretch of amino acids in the N-terminal could increase the potency of contractile effect of bombesin-like peptides [31]. Therefore, it seemed conceivable that the functional activity sites might be exposed and the half-life of peptides would be extended by binding the longer amino acid peptides to the receptors. These data indicated their abilities to bind to the mammalian receptors of both bombesin-OS and bombesin-PE, thus resulting in the activation of these receptors. 

Undoubtedly, the increasing discovery of the functional peptides in the skin secretions may give scientists a new way to improve the application of therapeutic agents and to develop drugs for human healthcare. Since the progress of molecular techniques, the nucleotide sequences of orthologous genes are well studied. In addition to the conventional research on the fossil record and morphological characteristics, new phylogenetic studies of the relationship between species may be able to provide a novel aspect, and may help in a deeper understanding amphibian evolutionary history.

## 4. Materials and Methods

### 4.1. Specimen Biodata and Secretion Harvesting

Specimens of Chinese piebald odorous frog, *Odorrana schmackeri* were captured during expeditions in Fujian, People’s Republic of China and European edible frog, *Pelophylax kl. esculentus*, were obtained from a local herpetological supplier. Adult frogs were settled in vivaria for 4 months prior to harvesting the secretions. Skin secretion was obtained from the dorsal skin using mild transdermal electrical stimulation as described previously. The stimulation-induced secretions were washed from the skin using de-ionized water, rapid frozen in liquid nitrogen, lyophilised and followed by storage at −20 °C until use. Sampling of skin secretion was performed under the UK Animal (Scientific Procedures) Act 1986, project licence PPL 2694, issued by the Department of Health, Social Services and Public Safety, Northern Ireland. Procedures had been vetted by the IACUC of Queen’s University Belfast, and approved on 1 March 2011.

### 4.2. “Shotgun” Cloning of Odorrana schmackeri and Pelophylax kl. esculentus Skin Secretion-Derived cDNA Library

Five milligrams of lyophilised skin secretion were dissolved in 1 mL of cell lysis/mRNA protection buffer that was obtained from Dynal Biotec, UK. Polyadenylated mRNA was isolated from this by magnetic oligo-dT Dynabeads according to the manufacturer’s instruction (Dynal Biotec, Merseyside, UK). The isolated mRNA were then subjected to 5′ and 3′-rapid amplification of cDNA ends (RACE) procedures to obtain the full-length DNA sequences of bombesin precursors using a SMART-RACE kit (Clontech, Palo Alto, CA, USA) as per manufacturer’s instructions. Briefly, a NUP (supplied with the kit) and a degenerate sense primer (S: 5′-CARAAYACITAYMGIGCICC-3′; R = A + G, Y = C + T, M = A + C) were used in the 3′-RACE reactions. PCR products were gel-purified and cloned using a pGEM-T vector system (Promega Corporation, Southampton, UK) and sequenced using an ABI 3100 automated sequencer.

### 4.3. Isolation and Structure Identification of Peptides

Five milligrams of lyophilised skin secretion were dissolved in 1 mL solution buffer (trifluoroacetic acid (TFA)/water = 0.05/99.95, *v*/*v*), and then the mixture was centrifuged. The supernatant was aspirated and followed by injection into a reverse phase HPLC column (Phenomenex C-18, 250 mm × 10 mm). Fractions were separated and collected by using a Cecil Adept 4200 HPLC system (Amersham Biosciences, Buckinghamshire, UK). A linear gradient elution was carried out using the mobile phase in which the composition was changed from water/TFA (99.95/0.05, *v*/*v*) to water/acetonitrile/TFA (19.95/80.00/0.05) over 240 min and the fractions were monitored at 214 nm. Samples (100 μL) were removed from each fraction in triplicate, lyophilised and stored at –20 °C. The molecular masses of peptides in the fractions were determined using matrix-assisted laser desorption/ionization, time-of-flight mass spectrometry (MALDI-TOF MS) on a linear time-of-flight Voyage DE mass spectrometry (Thermo Fisher Scientific, San Francisco, CA, USA) in positive detection mode using α-CHCA as the matrix.

### 4.4. Solid-Phase Peptide Synthesis of Bombesin-OS and Bombesin-PE Peptides

Fmoc solid phase synthesis was applied on a Tribute Peptide Synthesizer (Protein Technologies, Tucson, AZ, USA) to produce peptide replicates. 1.2 mmol of amino acids mixed with 1.2 mmol of HBTU were transferred to the reactor containing Fmoc-Cys (Trt)-Wang resin. The Fmoc group was deprotected by using piperidine (20% in DMF). The peptide bond coupling was activated and completed in 1 M 11% NMM in DMF. The peptides were cleaved from the resins using a trifluoroacetic acid-EDT-triisopropylsilane-H_2_O (TFA-EDT-TIS-H_2_O; 94:2:2:2) cocktail, and then the final peptide was purified by using reverse phase HPLC. The primary structure and purity of peptides were determined by MALDI-TOF MS and LCQ MS/MS fragmentation sequencing.

### 4.5. The Effects of Bombesin-OS and Bombesin-PE on Rat Smooth Muscles Tension

Female Wistar rats (250–300 g) were humanely killed by carbon dioxide asphyxiation based on institutional animal experimentation ethics and UK animal research guidelines. The smooth muscle tissues of bladder, uterus and ileum were gently pulled out and then immediately put into ice-cold Krebs’ solution (118 mM NaCl, 1.15 mM NaH_2_PO_4_, 2.5 mM CaCl_2_, 25 mMNaHCO_3_, 4.7 mM KCl, 1.1 mM MgCl_2_ and 5.6 mM glucose). The smooth muscle tension was determined by an isolated tissue bath assays. Briefly, the small tissue strips were immersed in Kreb’s solution bubbled continuously with 95% O_2_ + 5% CO_2_ (2 mL/min) at 37 °C for 10 min, and the muscle tension was recorded using a transducer (Neurolog 61, Digitimer Ltd., Welwyn Garden, UK). The bladder, uterus, artery, and ileum tissue were stretched, maintaining the normal physiological tension of 0.75 g, 0.5 g, and 0.5 g respectively. Bombesin-OS and bombesin-PE solutions, ranging from 10^−9^ M to 10^−3^ M, were made in Kreb’s solution and then added to the organ bath in a cumulative manner for at least 5 min before reaching the equilibrium. Then, the effects of these peptides on smooth muscles were determined using a tension sensor that is capable of detecting and recording the tension changes or changes in spontaneous contraction frequencies, followed by the amplification of the analog signal through a PowerLab System (AD Instruments Pty Ltd., Oxford, UK).

### 4.6. Statistical Anaylysis

Statistical analyses were performed using GraphPad Prism software (version 6.01, San Diego, CA, USA) and SPSS software (version 24, Chicago, IL, USA). Comparison between two groups was analysed using a two-tailed unpaired student t-test. Comparison between three groups was analysed using one-way ANOVA, with post hoc Turkey’s multiple comparisons test. Observed power was calculated with each analysis to confirm that the sample size was sufficient to support the data. A *p*-value less than 0.05 was considered significant.

## 5. Conclusions

In this study, two bombesin-like peptides, bombesin-OS (pGlu-NTYRAPQWAVGHLM-NH_2_) and bombesin-PE (pGlu-IPQWAVGHFM-NH_2_) were identified in the skin secretions of *Odorrana schmackeri* and *Pelophylax kl. esculentus*, respectively. The precursor of bombesin-OS was virtually identical to that of a bombesin-like peptide from *Odorrana grahami*, and the precursor of bombesin-PE, on the other, was highly identical to that of the bombesin-like peptide, ranatensin. Furthermore, according to BLAST analysis of the open-reading frame, bombesin-PE was shown to belong to the ranatensin subfamily. Both bombesin-OS and bombesin-PE were demonstrated to have the activities not only to increase the frequency of spontaneous contraction of rat uterus but also to moderate the stimulated contraction of rat bladder and ileum smooth muscles.

## Figures and Tables

**Table 1:** Multiple comparisons of contractile activity of peptides (1 µM) on isolated tissues. The error term is Mean Square (Error) = 0.002. *. The mean difference is significant at the 0.05 level.

	(I) Sample	(J) Sample	Mean Difference (I-J)	Std. Error	Sig.	95% Confidence Interval	
						Lower Bound	Upper Bound
Bladder	Bombesin-OS	Bombesin-PE	−1.107636 *	0.3938992	0.025	−2.088772	−0.126500
		V	1.027333 *	0.4119152	0.050	0.001323	2.053344
	Bombesin-PE	Bombesin-OS	1.107636 *	0.3938992	0.025	0.126500	2.088772
		V	2.134970 *	0.3810193	0.003	1.185915	3.084024
	V	Bombesin-OS	−1.027333 *	0.4119152	0.050	−2.053344	−0.001323
		Bombesin-PE	−2.134970 *	0.3810193	0.000	−3.084024	−1.185915
Uterus	Bombesin-OS	Bombesin-PE	0.244444	1.1355882	0.975	−2.584113	3.073002
		V	7.333333 *	1.1650889	0.001	4.431295	10.235372
	Bombesin-PE	Bombesin-OS	−0.244444	1.1355882	0.975	−3.073002	2.584113
		V	7.088889 *	1.1355882	0.000	4.260332	9.917446
	V	Bombesin-OS	−7.333333 *	1.1650889	0.000	−10.235372	−4.431295
		Bombesin-PE	−7.088889 *	1.1355882	0.000	−9.917446	−4.260332
ileum	Bombesin-OS	Bombesin-PE	−0.010141	0.0207680	0.877	−0.061748	0.041465
		V	0.182444 *	0.0217817	0.000	0.128319	0.236570
	Bombesin-PE	Bombesin-OS	0.010141	0.0207680	0.877	−0.041465	0.061748
		V	0.192586 *	0.0207680	0.000	0.140980	0.244192
	V	Bombesin-OS	−0.182444 *	0.0217817	0.000	−0.236570	−0.128319
		Bombesin-PE	−0.192586 *	0.0207680	0.000	−0.244192	−0.140980

## Abbreviations

The following abbreviations were used in this manuscript:

cDNA	Complementary DNA
RACE	Rapid Amplification of cDNA Ends
Fmoc	9-fluorenylmethyloxycarbonyl
α-CHCA	Alpha-cyano-4-hydroxycin-namic acid
HBTU	2-(1*H*-benzotriazol-1-yl)-1,1,3,3-tetramethyluronium hexauorophosphate
EDT	Ethanedithiol
NMM	N-methylmorpholine
DMF	Dimethylformamide
NUP	Nested Universal Primer
